# Dynamics of Health Care Financing and Spending in Serbia in the XXI Century

**DOI:** 10.3389/fpubh.2019.00381

**Published:** 2019-12-13

**Authors:** Kristijan Krstic, Katarina Janicijevic, Yuriy Timofeyev, Evgeny V. Arsentyev, Gvozden Rosic, Sergey Bolevich, Vladimir Reshetnikov, Mihajlo B. Jakovljevic

**Affiliations:** ^1^Center for Rehabilitation Medicine, University Clinical Center Kragujevac, Faculty of Medical Sciences, University of Kragujevac, Kragujevac, Serbia; ^2^Department of Social Medicine, Faculty of Medical Sciences, University of Kragujevac, Kragujevac, Serbia; ^3^National Research University Higher School of Economics, Moscow, Russia; ^4^Department of Public Health and Healthcare, First Moscow State Medical University (Sechenov University), Moscow, Russia; ^5^Department of Dentistry, Faculty of Medical Sciences, University of Kragujevac, Kragujevac, Serbia; ^6^Department of Human Pathology, First Moscow State Medical University of the Ministry of Health of the Russian Federation (Sechenov University), Moscow, Russia; ^7^Department of Global Health Economics and Policy, Faculty of Medical Sciences, University of Kragujevac, Kragujevac, Serbia

**Keywords:** health care, public health expenditure, private health expenditure, Serbia, Semashko model

## Abstract

Serbia is an upper-middle income Eastern European economy. It has inherited system of health provision and financing, which is a mixture of Soviet Semashko and German Bismarck models. So far, literature evidence on long-term trends in health spending remains scarce on this region. Observational descriptive approach was utilized relying on nationwide aggregate data reported by the Republic Health Insurance Fund (RHIF) and the Government of Serbia to the WHO office. Consecutively, the WHO Global Health Expenditure Database^1^ was used. Long-term trends were extrapolated on existing data and underlying differences were analyzed and explained. The insight was provided across two distinctively different periods within 2000–2016. The first period lasted from 2000 till 2008 (the beginning of global recession triggered by Lehman Brothers' bankruptcy). This was a period of strong upward growth in ability to invest in health care. Spending grew significantly in terms of GDP share, national and per capita reported expenditures. During the second period (2009–2016), after the beginning of worldwide economic crisis, Serbia was affected in a way that its health expenditure growth in PPP terms slowed down effectively fluctuating around plateau values from 2014 to 2016. Serbia health spending showed promising signs of steady growth in its ability to invest in health care. Consolidation marked most of the past decade with certain growth rates in recent years (2017–2019), which were not captured in these official records. The future national strategy should be devised to take into account accelerated population aging as major driver of health spending.

## Introduction

Since the beginning of the twenty-first century macroeconomic and political reforms, alongside with economy strengthening, led to rapid growth of health spending in Serbia (1). This trend has reached essentially a plateau level since the beginning of the global macroeconomic recession, recording fluctuations in 2008–2016 (2). These developments were led by several core-unfolding events across the nation. One of them was transformation of local pharmaceutical sector from domestic manufactured, generics-dominated one, toward imported brand-name and high-budget impact innovative medicines (3, 4). Acceleration of late stage population aging (5) was another far-reaching evolution with profound long-term impact on health financing sustainability, just like elsewhere throughout the Balkans (6), Eastern Europe (7, 8), and Asia (9, 10). Among other demand-side issues, sharply increased citizen welfare, purchasing power and living standards since the 1990s ultimately led to the increased civil expectations toward affordability of cutting-edge technologies (11). This fact has added pressure to the authorities to provide for their reimbursement in resource-constrained setting (12).

Last, but not least, epidemiologic transition of morbidity and mortality patterns taking place and accelerating since the post-WWII decades, brought upon blossoming of non-communicable diseases (13). Unlike mostly acute and today curable infectious diseases of the past (14), these were chronic, life-time expensive disorders (15). In the case of cancer (16), they have created so-called “the last year of life phenomenon” (17). This meant that the last 9 or 12 months of suffering and palliative care requiring expensive intensive care admissions (18) or oncology treatments (19), frequently equal the entire lifespan consumption of a citizen (20).

All these changes dictated the strong strive for health system reorganization (21) and advancement in terms of greater cost-effectiveness of resource allocation policies (22) and need for improved outcomes (23). Probably the most convincing success stories on adaptive responses to population aging challenge come from the similarly shaped post-communist health care sectors of some of the leading BRICS nations (BRICS is the acronym coined for an association of five major emerging national economics: Brazil, Russia, India, China, and South Africa) (24–27).

The three leading early historical establishments of health care financing and provision models in Europe, later to be embraced by their peripheral descendant cultures, were the German Bismarck model (1883), the British Beveridge (28) model (adopted in 1911) and the Soviet-Russian Semashko (29), established in the early 1930s (30). The Semashko model, actually pioneered universal right to health care free of charge worldwide (31). It was made possible, being a part of system of a centrally-planned economy with domination of specialized institutions (32). The Bismarck model is a market-oriented model with decentralization (contract model), where primary health care acts as a gatekeeper to the system (33). This model features effect-based payments, evidence-based medicine and the capacity (excessive) is visibly reduced (34).

In the Republic of Serbia, public government-led and health insurance fund RFZO-led spending, remains by far more relevant in comparison to private provisions for health care (35). The Law on Health Insurance of the Republic of Serbia administers compulsory and voluntary health insurance (36). The RHIF is responsible for providing and managing compulsory health insurance, while voluntary insurance can be provided by private insurance. However, just like elsewhere throughout Central and Eastern European post-Semashko health systems, it remains scarce and marginal contributor to the universal coverage of these markets (37–39).

The aim of this paper is to analyze the dynamics of health care financing and spending in Serbia in the twenty-first century. For this purpose, national and international databases were employed. The novelty of this study, compared to, e.g., (40) consists in shedding light on the healthcare financing in Serbia. Following this introduction, the overview of the country background and current Serbian national healthcare system are presented. Next, data and method are introduced. After this, the results are presented. Discussion precedes the concluding section.

## Healthcare Financing in Serbia

In Serbia, all the money directly or indirectly is provided by the citizens through financing the state budget, compulsory health insurance, direct payment “out-of-pocket,” financing from the community funds, donations, loans, etc. (21) The health care system in Serbia is funded through a combination of public finances and private contributions (41). The most important source of health care financing is the National Health Insurance Fund of the Republic of Serbia (42). Health Insurance Fund is financed also with supplementary financing from various budgetary sources, such as the Pension Fund, the Ministry of Finance Fund for Unemployed, etc. Funds for the health care of the insured persons are provided from the RHIF (40).

Due to the essential absence of private health care insurance (43), private funding is more or less completely based on out-of-pocket payments (44). It is supplemented by contributions from a small number of major companies, which have (and fund) their own institutions, specializing in the treatment of occupational diseases and provide primary care services (45). More than 90% of public costs are financed through the RHIF. In Serbia, about 69% of total current health expenditure (TCHE) are financed by public sources, thereof, the largest share by RHIF^2^.

An employee is granted a health insurance, which depends on employment status (temporary or permanent employment). Health insurance for retired persons is based on their paid contributions during the working life (46). The employer is obliged to pay contributions to the RHIFon a regular basis, and a health insurance notice with prolonged validity will be provided to the insured person (23). Unemployed persons have to possess mandatory employment notice with employment record. In addition, a citizen has to register at the National Employment Service based on somebody's place of residence (47). Lastly, applying for health insurance at branch offices of the RHIF, according to the place of residence, is required. Health insurance is without any payment due for unemployed persons registered at the National Employment Service (48).

Variety of countries worldwide, rich and poor alike, have historically experienced obstacles when it comes to ensuring adequate access and equity of medical care. Universal health coverage means that such care has to be accessible to the majority of citizens regardless of their income level, within the range of accessible assets (49). It was an additional challenge to ensure affordability of cutting-edge innovative medical technologies and evidence-based medicine (50). Serbia, being the historical core of the former Yugoslavia and the oldest constitutional monarchy of the Balkans, has centuries long statehood tradition (51). This has positively reflected in its evolving legislative framework in health care and successful overcoming of many hurdles since the 1990s (18).

## Current Serbia's National Health Sector Challenges

Upper-middle income Serbia shares core contemporary challenges within the WHO European Region consisting of 53 countries (52). Population aging, global macroeconomic recession as of 2008–2016, Middle Eastern (53) migration routes (54) and fiscal sustainability issues (55) are common to Eastern Europe (56) and Western EU15 nations (57).

The major factor in health deprivation is the Third Demographic Transition also known as population aging. This became global phenomenon in the second half of the twentieth century, named “The Silver Tsunami.” Based on the last 2011 Census, and according to all the characteristics of population aging, Serbian population can be classified in the group of very old populations, not only in Europe, but also globally (58). At the same time, the changes achieved in the last decades, especially by the end of the twentieth and the early twenty-first century, indicate that Serbia has been exposed to a very intense population aging. This process has been manifested in low and steadily declining share of the youth and high and continuously increasing share of the elderly in the total population of the country (59).

In Serbia, the 2011 Census registered 1,025 thousand of people under the age of 15. At the same time, there were 1,250 thousand of the elderly. This means that the number of the elderly, as in the previous 2002 Census, exceeded the number of young people. In <10 years, the number of young people decreased by 150 thousand, whereas the number of the elderly increased by 10 thousand people. The share of young people decreased from 15.8 to 14.3%, while the share of the elderly increased from 16.7 to 17.4%.

## Data and Method

This brief research report article represents the descriptive data analysis of macroeconomic and health expenditure indicators in Serbia in 2000–2016. The WHO Global Health Expenditure Database was selected as a core data source among the Serbian National sources, EuroStat, OECD Health, World Bank Health Data and others. It was employed due to its internationally comparable fiscal flows tracking across jurisdictions and countries due to the National Health Accounts system adoption by the UN agencies inclusive of the WHO^3^, back in 1995 and its consecutive revisions in 2000 and 2011. Core indicators of income and spending observed were: current health care expenditure expressed as a percentage of GDP and per capita in nominal US dollars and in purchase power parity (PPP), domestic general government health expenditure per capita in nominal US dollars and in PPP, domestic private expenditure per capita in nominal US dollars and in PPP, as well as out-of-pocket expenditure expressed in nominal US dollars and PPP, as collected by the WHO from national governments. Financial parameters are expressed in US currency, nominal, and PPP US dollars, for easier comparability with the majority of published literature sources.

## Results

**Figure 1:**
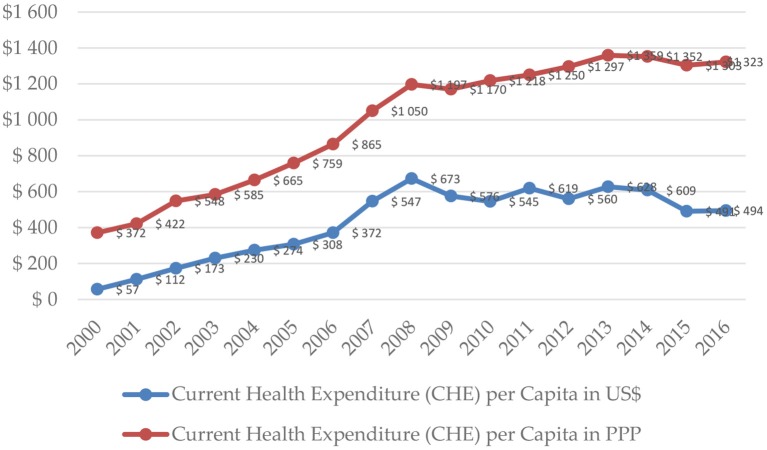
Current health care expenditure per capita in Serbia in 2000–2016. Source: WHO Global Health Expenditure Database.

**Figure 2:**
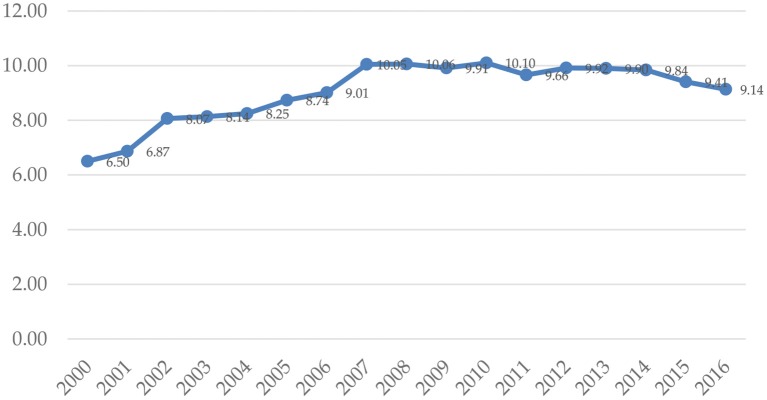
Current health expenditure as a percentage of GDP in Serbia in 2000–2016. Source: WHO Global Health Expenditure Database.

Expenditure for health care in USD per capita terms grew and later fluctuated in the period 2000–2016 in their absolute nominal and PPP amounts (Figure 1). In nominal and PPP dollar terms alike, health care expenditure rose, along with the stable share in GDP, after an increase in 2007. Nevertheless, in 2009, due to a slower growth of health care expenditure and negative growth of GDP, the total health care expenditure per capita in nominal dollar terms was inferior to the respective figure from the year before. In 2007, the share of total health care expenditure in GDP terms increased by one percentage point and stabilized in the following years at solid 10%, with a slight increase to 10.1% in 2010 (Figure 2). However, in the recent years, data indicates that there was a considerate decrease to 9.41% in 2015. The share of health care public expenditure in GDP was 9.14% in 2016 with a similar changing pace in the examined period.

**Figure 3:**
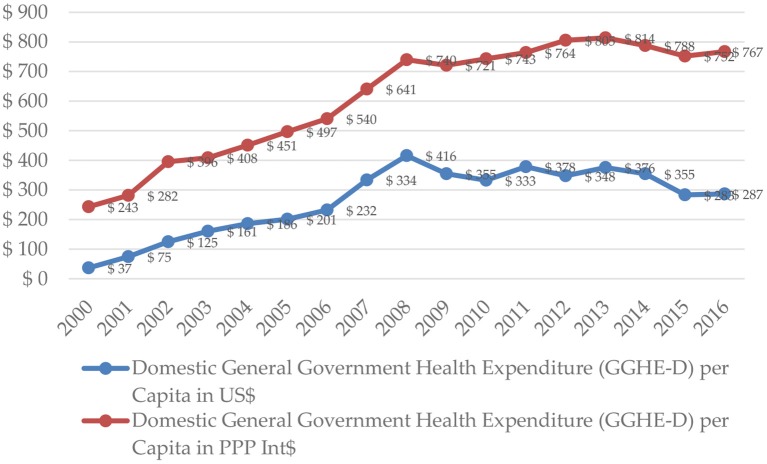
Domestic general government health expenditure per capita in Serbia in 2000–2016. Source: WHO Global Health Expenditure Database.

**Figure 4:**
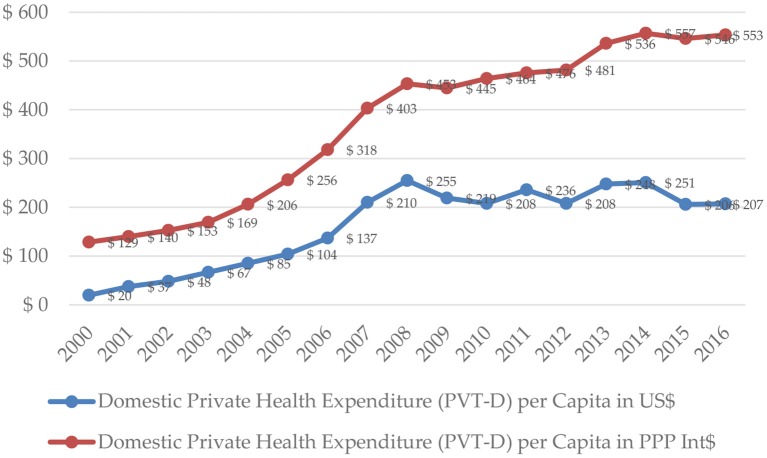
Domestic private expenditure per capita in Serbia in 2000–2016. Source: WHO Global Health Expenditure Database.

In 2010, the total health care expenditure in Serbia stabilized at the level of $545 ($1,218 PPP) per capita. During the next 3 years (2011-2013), level of total health expenditure improved to $628 ($1,359 PPP) in 2013. During the last years, there was a significant contraction to $494 ($1,323 PPP) in 2016. In 2016, the expenditure, incurred by the Domestic General Government Fund, was $287 ($767 PPP) per capita (Figure 3), while the domestic private expenditure for health care was equivalent to $207 ($553 PPP) per capita (Figure 4).

When it comes to allocations for health care expressed as percentage of GDP, Serbia is slightly above the average of the European Union, with its 9.03%, i.e., approximately at the level of Belgium, Austria, Greece, and Bosnia and Herzegovina (60). In comparison to the EU countries, some non-EU countries and the average spending in the EU countries, the Republic of Serbia allocates rather modest absolute amount of funds for health care (61).

Predictably, out-of-pocket expenditure per capita almost replicate the trends demonstrated by the domestic private expenditure for health care, both in nominal and PPP dollar terms.

## Plausible Causes and Recommended Policies

Health reform in Serbia started in 2003 and still is undergoing. This reform should focus on bringing health system to the optimal functionality, so it can deliver the highest positive effect on health status of the population, equity in using and financing the health care system and constant improvement in financial sustainability of the system (62, 63).

The government should accept that one of the core axioms of a country's stable development must be health of the nation (64). Poor health is in direct correlation with economic productivity (65) and development (66). Other notable factor that should be brought into focus is decentralization. The core functionality of the health care system is to be transferred while primary health care remains a top priority (67). The additional funding allocations from local level to the second administrative subdivisions of Serbia or municipalities should be mobilized (68). It appears that health system would benefit from limiting influence of frequent governments' (69) and political changes (70). Besides, municipalities and health administrative regions could share the financial risks more equally (71).

Modernizing health information system since reliability and timely information are mandatory for the health care system reform (72), represents the solid base on which planning, decision-making, managing, monitoring and implementation of reforms can be done (1). Invention of the information-communication system serving for managing the health care system (73) and gathering of all information system networks established by health institutions (74), insurance funds and regional centers (75) would tremendously aid in all of the mentioned key factors to health care system reform (76–78).

## Discussion

Since the end of the Cold War Era, Serbia has suffered the painful political and economic transformation from a centrally-planned socialist economy to a free market-driven capitalist one. It has entered the so-called “transitional period” with a decade-long delay due to the civil wars of the former Yugoslavia, the consecutive sanctions and poverty, while Serbs were a major refugee ethnicity in Europe (79–81). Economic recovery allowing for increased investment in health care and social policies took place effectively since 2000, which is exactly the period of this research (82).

Degree of socio-economic development, efficiency of fund raising and priority agenda of health care among policy-makers, are some of the core drivers of local financial sustainability. Existing resources could be re-allocated in a way that will improve medical services delivery and provision. Classical inefficiencies, such as lengthy hospital admissions (83), long waiting lists for the major surgeries or invasive radiology interventions, drug shortages (84) or unaffordability of cost-effective targeted medicines (12), might become a matter of the past to a large degree. So far negotiated prices for curative technologies and preventive measures with the major industry suppliers and large hospitals, are surpassing the available Republican Health Insurance Fund's budget, creating annual net losses.

Essentially, all of the late nineteenth and the early twentieth century European health systems were built on a demographic growth model (85) with epidemiology burden of that time (86) being dominated by infectious diseases, traumatism, neonatal, and maternal mortality (87). Today, in 2019, given the radically different circumstances in European continent, it is obvious that they should all have to be changed from the very basement to the very top of its hierarchy. Given the disposable real GDP of Serbia and its share allocated to health care, authorities, and public opinion become aware of necessity for profound and long-term change.

## Data Availability Statement

Publicly available datasets were analyzed in this study. This data can be found here: https://apps.who.int/nha/database, https://www.who.int/health-accounts/en/.

## Author Contributions

MJ, VR, EA, and SB: conceptualization. MJ and VR: methodology and resources. VR, YT, and MJ: validation. KK, KJ, and EA: formal analysis, investigation, and data curation. MJ, KK, and KJ: writing—original draft preparation. YT and GR: writing—review and editing and visualization. MJ: supervision and project administration.

### Conflict of Interest

The authors declare that the research was conducted in the absence of any commercial or financial relationships that could be construed as a potential conflict of interest.

## Footnotes

^2^The National Health Insurance Fund of the Republic of Serbia. Available online at: https://www.eng.rfzo.rs/index.php/aboutus

^3^WHO National Health Accounts. Available online at: https://www.who.int/health-accounts/en/
